# A Novel Widespread Cryptic Species and Phylogeographic Patterns within Several Giant Clam Species (Cardiidae: *Tridacna*) from the Indo-Pacific Ocean

**DOI:** 10.1371/journal.pone.0080858

**Published:** 2013-11-20

**Authors:** Thomas Huelsken, Jude Keyse, Libby Liggins, Shane Penny, Eric A. Treml, Cynthia Riginos

**Affiliations:** 1 The University of Queensland, School of Biological Sciences, St Lucia, Australia; 2 Charles Darwin University, Research Institute for Environment and Livelihoods, Casuarina, Australia; 3 University of Melbourne, Department of Zoology, Melbourne, Australia; University of Texas, United States of America

## Abstract

Giant clams (genus *Tridacna*) are iconic coral reef animals of the Indian and Pacific Oceans, easily recognizable by their massive shells and vibrantly colored mantle tissue. Most *Tridacna* species are listed by CITES and the IUCN Redlist, as their populations have been extensively harvested and depleted in many regions. Here, we survey *Tridacna crocea* and *Tridacna maxima* from the eastern Indian and western Pacific Oceans for mitochondrial (*COI* and *16S*) and nuclear (*ITS*) sequence variation and consolidate these data with previous published results using phylogenetic analyses. We find deep intraspecific differentiation within both *T. crocea* and *T. maxima*. In *T. crocea* we describe a previously undocumented phylogeographic division to the east of Cenderawasih Bay (northwest New Guinea), whereas for *T. maxima* the previously described, distinctive lineage of Cenderawasih Bay can be seen to also typify western Pacific populations. Furthermore, we find an undescribed, monophyletic group that is evolutionarily distinct from named *Tridacna* species at both mitochondrial and nuclear loci. This cryptic taxon is geographically widespread with a range extent that minimally includes much of the central Indo-Pacific region. Our results reinforce the emerging paradigm that cryptic species are common among marine invertebrates, even for conspicuous and culturally significant taxa. Additionally, our results add to identified locations of genetic differentiation across the central Indo-Pacific and highlight how phylogeographic patterns may differ even between closely related and co-distributed species.

## Introduction

Giant clams of the genus *Tridacna* are among the most conspicuous marine invertebrates on coral reefs due to their large size and brilliantly colored mantle that contains photosynthesizing symbionts. Giant clams have traditionally provided raw material for tools, containers, and ornaments [1], and many populations are harvested for meat, shells, and the ornamental aquarium trade [2], [3]. Despite local management efforts, including mariculture [3], wild stocks of giant clams are depleted and some species are locally extinct in many areas of Southeast Asia and the South Pacific [3]–[5]. Consequently, most *Tridacna* species are listed by CITES (Appendix II)[6] and the IUCN Redlist [7].

There are currently eight [8] described species within the genus *Tridacna* (*T. crocea* Lamarck, 1819, *T. derasa* (Röding 1798), *T. gigas* (Linnaeus 1758), *T. maxima* (Röding 1798), *T. mbalavuana* Ladd, 1934, *T. rosewateri* Sirenko and Scarlato 1991, *T. squamosa* Lamarck 1819, and *T. squamosina* Sturany 1899), differentiated by morphology and habitat preference [9]–[12]. *Tridacna squamosina*, *T. rosewateri*, and *T. mbalavuana* have restricted distributions (Red Sea, Mauritius, and Fiji to Tonga, respectively), whereas *T. derasa, T. gigas, T. crocea, T. squamosa* and *T. maxima* are widely distributed in the Indian and Pacific Oceans, with the latter two extending their distribution into the Red Sea [8], [9]. Molecular phylogenetic investigations support monophyly of the described species [13]–[15], albeit with some disagreement among species relationships. An unpublished Master's thesis [16] also reports a morphologically distinct clam from Taiwan and uses mtDNA loci to show that this clam is highly divergent from sympatric *T. maxima*, potentially indicative of an additional unnamed species.

**Figure 1:**
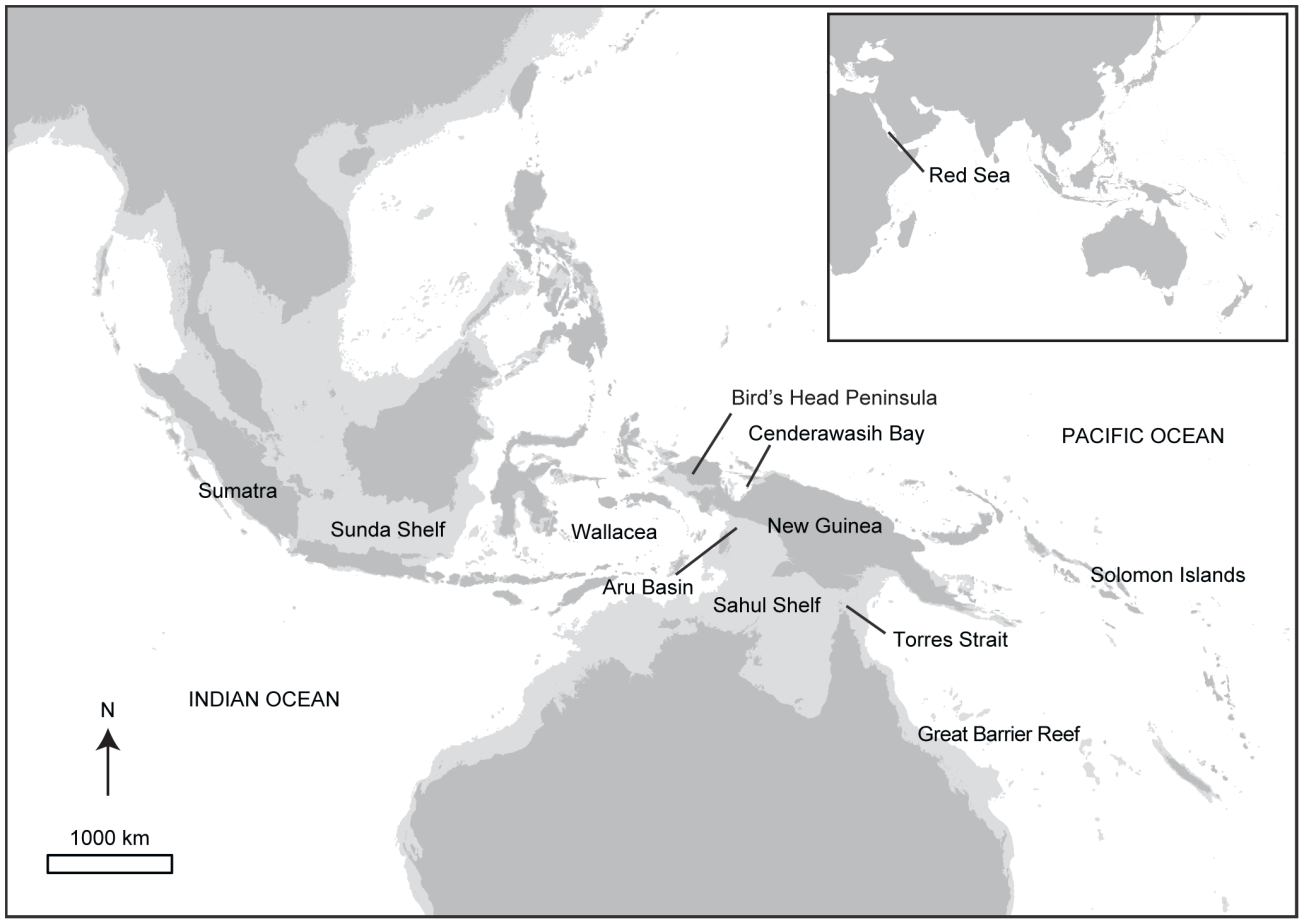
Study region. The light grey outline represents the lowest Pleistocene sea level (120 m depth contour).

The juncture between the Indian and Pacific Oceans (Fig. 1), where several species of *Tridacna* are sympatric [8], is a well-known epicenter of tropical marine biodiversity [17], [18]. Genetic surveys in this region have revealed cryptic species, even among conspicuous and well-studied marine invertebrates [19], [20]. Many species show substantial intraspecific genetic division between the ocean basins (reviewed by [21]), with the Sunda Shelf, Molucca and Flores Seas, Makassar Strait, and Bird's Head region of northwest New Guinea emerging as locations of genetic discontinuities [21], [22]. These locations span the archipelago commonly referred to as Wallacea, which falls between the Sunda (southeast Asia) and Sahul (Australia and New Guinea) continental shelves and was the only point of permanent oceanic connection between the Indian and Pacific Oceans throughout the Pleistocene [23].

Phylogeographic and population genetic surveys have intensely sampled *T. maxima* and *T. crocea* throughout Wallacea using mitochondrial (mtDNA) markers [24]–[26], allozymes [27], [28], and microsatellites [29]. Both *T. crocea* and *T. maxima* have been shown to contain distinct mtDNA clades associated with Sumatra (Sunda), Wallacea, and northwest New Guinea (Sahul, particularly in Cenderawasih Bay) [24]–[26]. These lineages are sympatric in some populations, for instance *T. maxima* from northern Java has both Sumatran and Wallacean mitotypes, and similarly *T. crocea* populations from Halmahera eastward through Cenderawasih Bay contain both Wallacean and northwest New Guinean lineages [26], [29]. Microsatellite genotyping of *T. crocea* corroborates the distinctiveness of Sumatran and Cenderawasih populations, with evidence for mixing in Wallacea of local genotypes with Cenderawasih-like genotypes [29]. Thus, substantial genetic differentiation typifies at least two *Tridacna* species in this region.

In the Pacific Ocean, *T. derasa, T. gigas, T. maxima* and *T. crocea* have been genetically surveyed, primarily with allozyme markers [27], [28], [30]–[35], but also with mtDNA [36]. These studies show genetic divisions between western and central Pacific populations but with some indication that eastern Australian populations show greater affinities with Philippine populations than they do with other western Pacific populations [30], [33]. Great Barrier Reef populations (eastern Sahul) form a cluster distinct from, but closely related to, Philippine populations for *T. maxima* and *T. derasa* but with low sampling in the Philippines (two and one populations, respectively) and no sampling in Wallacea or Sunda regions. Thus, it is unknown whether substantial genetic divergence reflects the geographic distance separating the Philippines and eastern Sahul or is indicative of distinct regional groupings.

Here, we examine DNA sequence diversity of *T. crocea* and *T. maxima* whose sampled distributions include the eastern Indian Ocean, Wallacea, and western Pacific Oceans. Data from new samples, predominantly from the western Pacific, are merged with data from previous studies, especially from Wallacea (e.g. [24], [25], [26]), to present a unified summary of phylogeographic patterns and a point of contrast to earlier broadscale studies based on allozymes [30], [32], [33], [35]. We use phylogenetic analyses to assess evolutionary relationships among species and also gauge regional geographical divisions within species.

## Materials and Methods

### Sampling and permits

Small mantle biopsies were non-lethally collected from animals with morphology characteristic of *Tridacna maxima* and *T. crocea* at 0–20 m depth from the Solomon Islands, and in Australia from Ningaloo Reef, Heron Island, Lizard Island, the Torres Strait and Lihou Reef. All sampling and tissue transport was in accordance with local and international regulations. Permit details are as follows: Lihou Reef, Australia: Department of Sustainability, Environment, Water, Population & Communities (Access to Biological Resources in a Commonwealth Area for Non-Commercial Purposes permit number: AU-COM2008042); Lizard Island and Heron Island, Australia: Great Barrier Reef Marine Park Authority and Queensland Parks and Wildlife (Marine Parks Permits: G08/28114.1, G09/31678.1, G10/33597.1, G11/34640.1); Ningaloo Reef, Australia: Western Australia Department of Environment and Conservation (License to take Fauna for Scientific Purposes: SF007126, SF006619, SF008861; Authority to Enter Calm Land/or Waters: CE002227, CE002627, Department of Fisheries, Western Australia Exemption 2046); Queensland: Queensland Government Department of Primary Industries (General Fisheries Permits: 118636, 150981); Torres Strait Islands, Australia: Commonwealth of Australia Torres Strait Fisheries Act 1984 and Australian Fisheries Management Authority (Permit for Scientific Purposes: 8562); Solomon Islands: Solomon Islands Government Ministry of Education and Human Resource Development and Ministry of Fisheries and Marine Resources (research permit: to S Albert, expiry 31/10/2011); Solomon Islands Government Ministry of Environment, Conservation and Meteorology (Convention on International Trade in Endangered Species of Wild Fauna and Flora export permit: EX2010/102); Australian Government Department of the Environment, Water, Heritage and the Arts (Convention on International Trade in Endangered Species of Wild Fauna and Flora import permit: 2010-AU-616020); Australian Quarantine Inspection Service (Permit to Import Quarantine Material: IP10017966).

### DNA sequences

DNA was extracted using a modification of the Qiagen DNeasy protocol [37]. Primers that targeted mitochondrial cytochrome oxidase 1 (*COI*) [24], [26], [38] and ribosomal *16S* [39] were used to amplify 390 and 417 basepair segments of the respective gene regions. A subset of samples were amplified for the partial nuclear *18S* and *ITS1* region (referred to as *ITS* in text) to provide independent estimates of phylogenetic relationships using primers from [13], [40]. PCR products were purified following a standard Exo-Sap protocol (New England Biolabs) and were sequenced by Macrogen (Korea). Trace files were edited in CodonCode Aligner (ver. 4.0.3). In addition, the NCBI repository of nucleotide sequences was searched for all published *Tridacna COI* and *16S* sequences (August 2012) representing both intraspecific [24]–[26], [41] and interspecific [9], [15], [16] surveys. These sequences were manually aligned [42] against our new sequences and against outgroups (*Hippopus hippopus, Hippopus porcellanus, Cerastoderma glaucum, Fragum sueziense*, and *Corculum cardissa*) and trimmed to a common length. For *ITS* there were several insertions/deletions that could not be reconciled, so these areas of low overlap were masked and not used for phylogenetic analyses.

### Phylogenetic analyses

Previous mtDNA surveys have used either *16S* [9], [15], [26] or *COI* [24]–[26], [41] gene regions. To unify these sources of data and address interspecific relationships, we initially took representative sequences across studies and linked them by our samples for which both gene regions had been sequenced in a concatenated search. For samples with only a single gene region (that is, sequences acquired from NCBI), information from the missing gene region was treated as missing data. Up to four individuals per species were retained representing the diversity of their species clade and prioritizing individuals with both *16S* and *COI* sequenced. Using StarBEAST v. 1.6.2 [43] each mtDNA gene region was treated as a separate partition. A general time reversible model with gamma distributed and invariant sites (GTR+G+I) was applied to each gene, with additional partitioning by codon position (1+2, 3) for *COI*. A relaxed molecular clock with an uncorrelated lognormal mutation rate was used for each gene. The *COI* and *16S* gene trees were linked, as mtDNA is a single linked locus (i.e. concatenated gene regions). Priors were set for nodes defining species as a log normal date (mean  = 0, SD = 1) with an offset representing the most recent estimate of the earliest fossil (*T. crocea*: 1.8, *T. maxima*: 5.3, and *T. squamosa*: 1.8 million years). The root of the Tridacninae was set as normal with mean date of 14 and SD of 2.5 million years. All fossil dates were based on [15], [44]. Speciation was modeled both as birth-death and Yule processes in independent runs of 250 million steps, with a burn-in of 25%, and yielded similar results.

Additional genealogical searches were performed using MrBayes ver. 3.1.2 [45] and RAxML (Randomized Axelerated Maximum Likelihood, Blackbox interface) [46]. Using the concatenated file of the same mtDNA sequences as above, searches were partitioned such that *16S* formed one partition, and *COI* formed a second partition with third codon positions partitioned separately from first and second (1+2, 3) for *COI*. In MrBayes, a GTR+G+I (nst  = 6, invgamma) model for all three partitions was used, with a search length of 10 million steps, sampling every 10,000 steps, and a burn-in of 25% (2.5 mill steps). Similarly, the GTR+G+I models were applied to these partitions in RAxML in a maximum likelihood search with 100 bootstrap replicates.

Locus-specific genealogies were also inferred for *COI*, *16S*, and *ITS* using both MrBayes and RAxML. Total data sets for each locus were assembled from all available sequences and then simplified by removing any identical haplotypes. Searches were performed under the same conditions previously described for *16S* (no partitions) and for *COI* (1+2, 3) with four separate searches of 10 million steps and the final 25% percent of trees retained (effectively a burn-in of 7.5 million steps). Search conditions for the partial nuclear *ITS* sequences were as above with indels treated as missing data and no partitioning.

The software Figtree (Rambaut: http://tree.bio.ed.ac.uk/software/figtree/) was used to assist with tree visualization and graphics preparation.

### Phylogeographic patterns

Intraspecific phylogeographic patterns were assessed by examining all available *COI* and *16S* sequences for *T. crocea*, *T. maxima*, and the distinct clade (*Tridacna sp.*) identified in the previous analyses. For each species-locus combination, a heuristic maximum parsimony search was conducted in PAUP* [47]. Because frequencies of published haplotypes are not consistently available, it was not possible to conduct standard population genetic analyses such as measures of diversity and differentiation. For intraspecific parsimony searches, the maximum number of trees was set to 1000 in PAUP*[47].

## Results

### DNA sequences

New DNA sequence data was generated for individuals from five locations (including 55 *COI*, 65 *16S*, and 50 *ITS* sequences: Genbank Acc. Nos. JX974838-JX975007). Combining these new sequence data with previously published data yielded aggregations of 405 *COI*, 132 *16S*, and 50 *ITS* sequences for *Tridacna* species, with 335 unique haplotypes for *COI* and 54 unique haplotypes for *16S*. In the new data generated for this study nearly all included individuals were sequenced for both *COI* and *16S* allowing us to link results from these two loci and provide a common context for the aggregated sequences from previous studies. Similarly, *ITS* sequences were obtained from an overlapping subset of individuals sequenced for *COI* and *16S*. Nexus files have been deposited in Treebase (http://purl.org/phylo/treebase/phylows/study/TB2:S13501).

### Phylogenetic analyses

**Figure 2:**
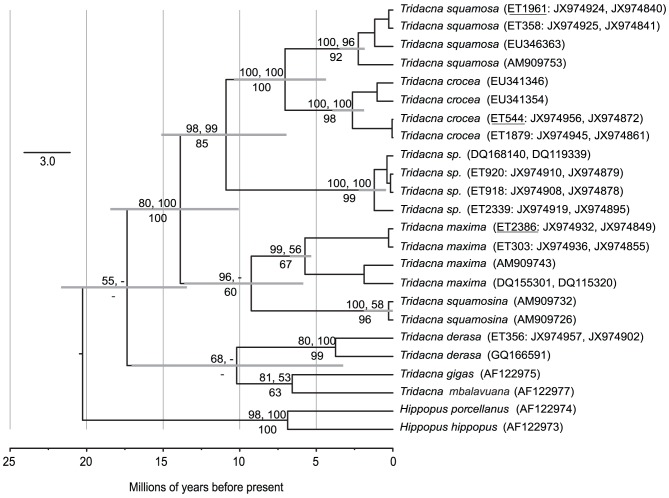
Species relationships within *Tridacna* based on concatenated mitochondrial DNA (*COI* and *16S*) sequences. The topology shown is a time calibrated maximum clade credibility tree inferred with StarBEAST under a birth-death model. Bayesian posterior probabilities from StarBEAST and MrBayes are above branches and RAxML bootstrap support percentages are below branches. Individuals with two accession numbers include both *COI* and *16S* sequences. Individuals that are underlined also appear in Fig. 4.

**Figure 3:**
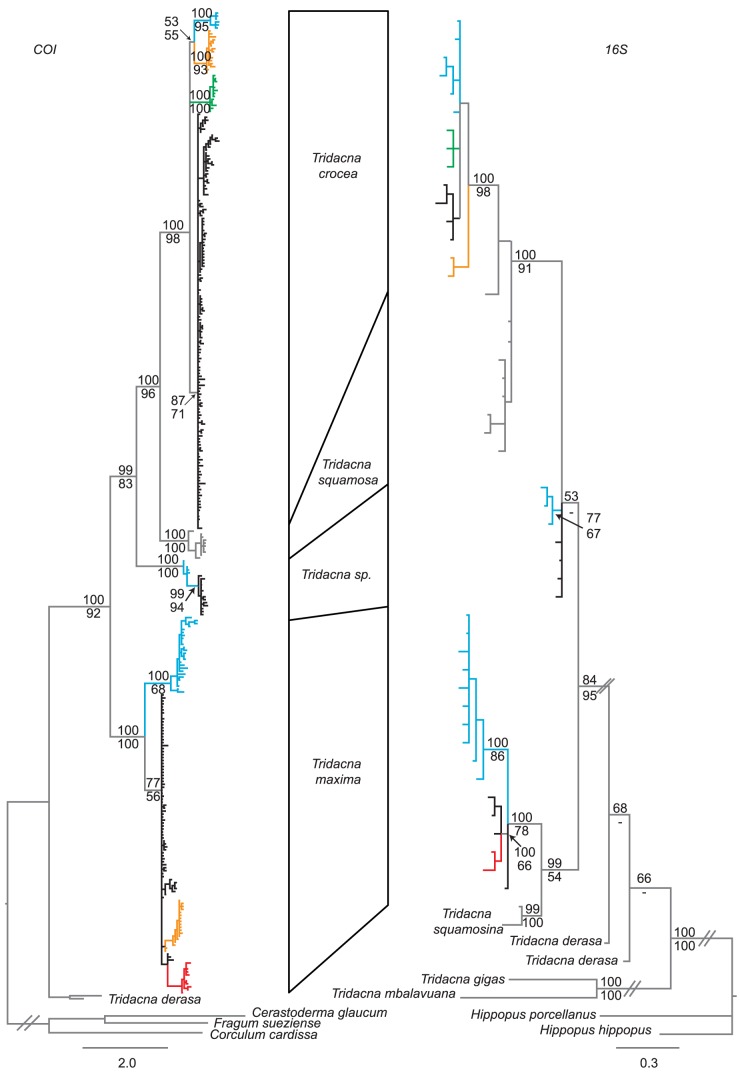
Bayesian phylogenetic trees for mitochondrial *CO1* and *16S*. MrBayesian consensus trees constructed for each gene region using all available data. Although different species and regions have differential representation, the two gene trees are concordant, as is expected for linked loci. Thus, overall patterns are consistent among research groups. Branch colors correspond to distinct lineages whose geographic distributions are described in Fig. 5.

**Figure 4:**
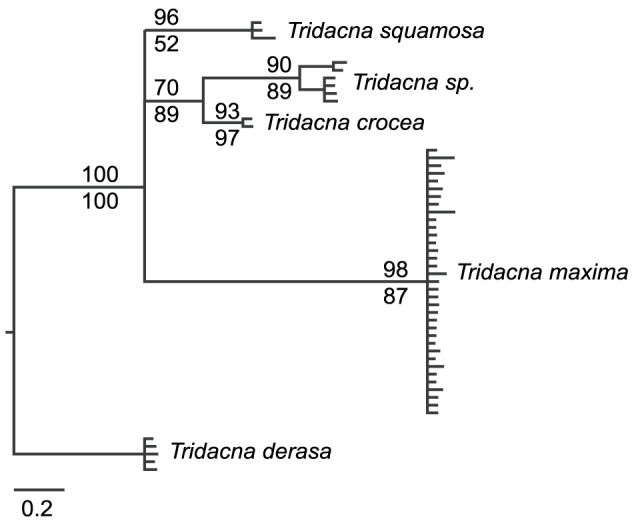
Species relationships within *Tridacna* based an *ITS* MrBayes consensus tree. Unalignable regions have been excluded. Bayesian posterior probabilities are above branches and RAxML bootstrap support percentages are below branches.

Phylogenetic analyses resulted in well-resolved topologies defining several clades within *Tridacna*. Tree topologies for the concatenated and single gene datasets were similar (Figs. 2–4), providing evidence for a robust and consistent phylogenetic signal. The concatenated analyses of mitochondrial *COI* and *16S* loci (Fig. 2) strongly support monophyly of *T. squamosa, T. crocea*, and a previously undescribed clade (but reported in [16]) formed well-supported terminal taxa, with more modest support for the monophyly of *T. maxima*. This undescribed clade (which we refer to as *Tridacna sp*.) was also well supported in single gene analyses of *COI* and *16S* (Fig. 3) and *ITS* (Fig. 4). *T. sp.* sequences were evolutionarily distinct from other species; the average pairwise *COI* sequence divergence between *T. sp* and *T. crocea* was 14.4% and was 12.6% between *T. sp*. and *T. squamosa*, as compared to 9.5% between *T. crocea* and *T. squamosa* (uncorrected pairwise distances).

Gene trees for *COI* and *16S* show concordant relationships among species (Fig. 3), confirming that independent research groups have sampled similar genotypes. The notable exception to the consistency across studies was the *16S T. derasa* sequence from [15] which did not cluster consistently with our *16S T. derasa* sequence (specimen ET358) even though our *COI* sequence from this same individual clustered with other *T. derasa* sequences including GQ166591 from [48]. For this reason, the *T. derasa* sequence from [15] was retained in the *16S* tree, but excluded from the joint *COI* and *16S* searches. All mtDNA-based genealogies supported *T. squamosa* and *T. crocea* as sister species (Figs. 2 and 3) whereas *ITS* based analyses gave modest support for *T. sp*. and *T. crocea* as sister species (Fig. 4). Within the mtDNA-based analyses, *T. derasa*, *T. gigas*, and *T. mbalavuana* appear consistently as basal lineages within *Tridacna* (Figs. 2 and 3). (No *ITS* sequences were available for these taxa.)

### Phylogeographic patterns

**Figure 5:**
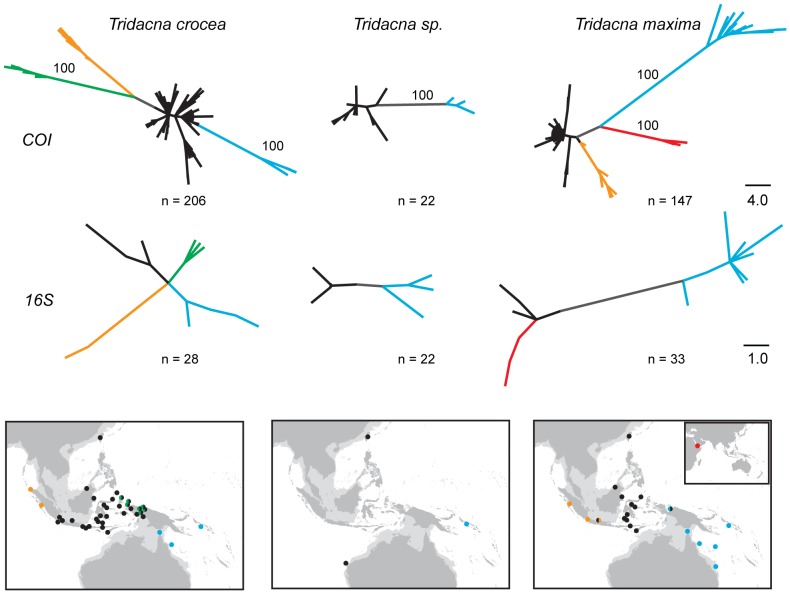
Unrooted parsimony trees and sampling locations for *Tridacna crocea*, *Tridacna sp*., and *Tridacna maxima*. Major lineages on networks are colored and the geographic extent of each lineage is indicated on the map. Relative frequencies of each haplotype are not depicted; each haplotype is shown in equal size (see text). Dots on maps indicate sampling locations and locations with two distinct sympatric lineages are shown as bisected circles (not indicative of relative frequencies). Support for monophyly of major clades among *COI* trees is based on 100 percent consistency of each branch among all equally parsimonious trees (a randomly chosen tree is depicted). Among *16S* trees, the single most parsimonious tree for *T. sp*. and *T. maxima* are shown, and for *T. crocea* both green and blue lineages were present in all six equally parsimonious trees. Colors indicate geographic locations of haplotypes and internal branches are in gray.

Within *T. crocea* and *T. maxima*, there was broadscale phylogeographic concordance of mtDNA gene trees (as shown in Fig. 5). *T. crocea* and *T. maxima* haplotypes from the Solomon Islands, the Torres Strait and Lizard Island (and additionally western New Guinea/Cenderwasih Bay, Lihou Reef and Heron Island for *T. maxima*) formed a distinct monophyletic ‘Pacific’ group (colored blue in Fig. 5). Sequences from the Sunda Shelf formed a second monophyletic group (colored orange in Fig. 5) as described in the original publications [24]–[26], although the location or the genetic break differed slightly for each species. Finally, sequences from Indonesia, Singapore, western New Guinea/Cenderwasih Bay and Taiwan formed a third group (black in Fig. 5). Most sequences published in Genbank are not georeferenced. We were, however, able to deduce the distinct clades typifying major regions from previously published surveys by recreating previously published analyses; *T. crocea* (yellow haplotypes of [24], grey clade of [26]) are shown in green and orange respectively, and *T. maxima* (yellow haplotypes of [25]) are shown in blue and orange respectively in Fig. 5.

For *T. maxima*, the northwest New Guinea clade formed a cluster with the Pacific clade, although no haplotypes were shared between the two locations. For *T. crocea*, however, haplotypes from northwest New Guinea and the western Pacific were members of two distinct monophyletic groups: the Pacific (blue) and the Wallacea (black) groups (Fig. 5). The *T. crocea* and *T. maxima 16S s*equences from [15], described as having been obtained from individuals sourced from aquarium stores, both fell within Pacific haplotype groups, suggesting that these purchased specimens had a Pacific origin.

Despite the reduced sampling for *T. sp*., a ‘Pacific’ lineage was similarly positioned in the Solomon Islands, and a distinct lineage, comprising samples from western Australia and Taiwan, geographically overlapped with the Wallacea (black) lineage portrayed in *T. crocea* and *T. maxima*. Similar phylogeographic patterns were evident for *COI* and *16S* for each species despite only partially overlapping sets of individuals forming the basis for each tree.

## Discussion

Despite their distinct shell morphology and longstanding cultural and commercial significance, our data reveal cryptic diversity within giant clams. Here, we find a previously undescribed clade of *Tridacna* (*Tridacna sp*.). This clade is supported by both mtDNA and nuclear gene regions (Figs. 2–4), which identify it as a unique, evolutionarily significant unit [49] with reference to previously described species. Our molecular phylogenetic analyses place *T. sp*. as a sister clade to *T. squamosa* and/or *T. crocea*, but in no instance was a close relationship between *T. sp*. and *T. maxima* suggested in our gene trees. Thus, molecular data do not support *T. sp.* being a variety of *T. maxima* as was suggested by Tang [16]. Clams with *T. sp*. mitotypes were found both at Ningaloo Reef in western Australia and in the Solomon Islands. Although only *T. sp.* and *T. squamosa* were identified among our clam samples from Ningaloo, it is likely that *T. maxima* also occur at Ningaloo (Penny unpub., [50]), and we found *T. sp.* sympatric with *T. maxima* and *T. crocea* in the Solomons.

**Figure 6:**
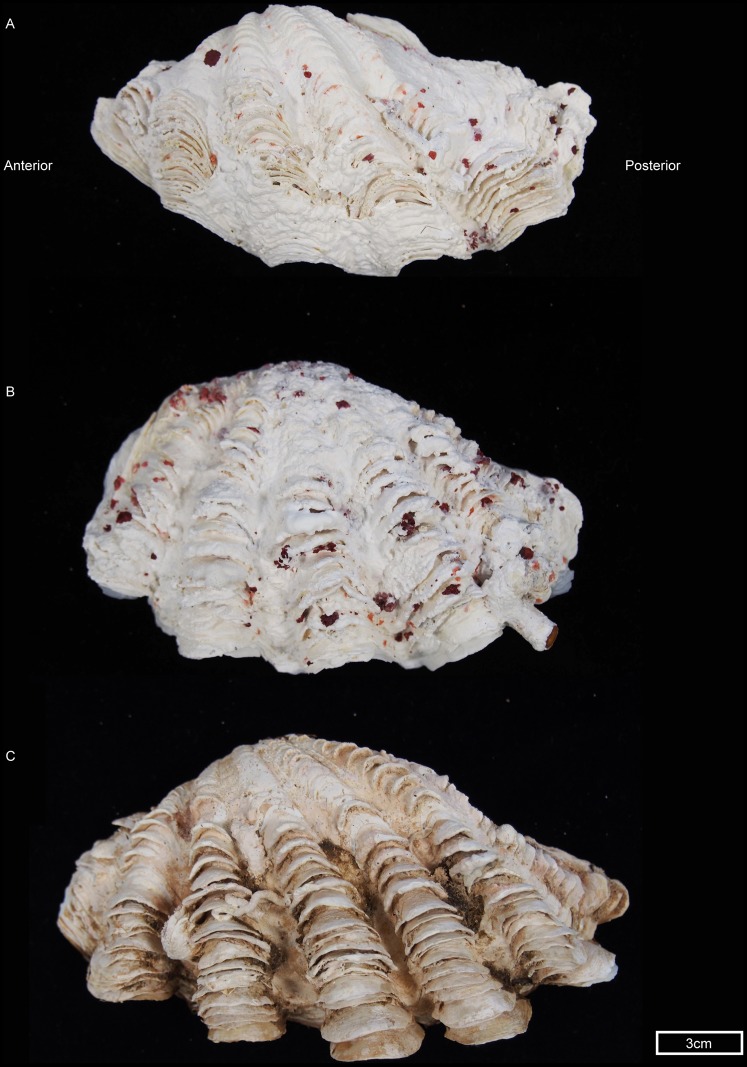
An individual with *Tridacna sp.* mtDNA demonstrating valve morphology consistent with *Tridacna maxima*. A) *Tridacna maxima* from Hibernia Reef, WA, Australia. Accession No# P.52722 (Museum Art Gallery Northern Territory (MAGNT)), original identification based on morphology, B) *Tridacna sp.* from Five Finger Reef, South of Coral Bay, Ningaloo Marine Park, WA, Australia. Accession No#. P.51911 (Museum Art Gallery Northern Territory (MAGNT)), C) *Tridacna maxima*, from north western WA, Australia, unregistered (Museum Art Gallery Northern Territory (MAGNT)). Photo credit: Shane Penny.

The *T. sp.* clade includes the single haplotype (*COI* and a *16S*) described from Taiwan [16]. Tang *et al.* (2005) suggested that there are morphological differences between *T. sp.* and *T. maxima*, including mantle pattern, shell lip shape, posterior adductor weight and the position of the incurrent aperture. Qualitative examination of an individual from Ningaloo Reef with *T. sp*. mtDNA shows shell characters typical of *T. maxima*: asymmetry of the valve with posterior elongation and dense rows of scales on folds (Fig. 6). *T. maxima* is well known for its morphological variability [51] and thus it is possible that previous morphological examinations of *T. sp* may have been identified it as *T. maxima*. (Additional morphological samples are not presently available as most collecting permits only allow non-lethal sampling of giant clams.) Our findings, therefore, lend support to Tang's conclusion that *T. sp.* is an undescribed species but we show that, rather than being a narrow-range endemic (such as *Tridacna rosewateri* from Mauritius [10]), *T. sp.* is widely distributed. Although it is not possible at present to delineate the distribution of *T. sp.*, it seems probable that *T. sp*. occurs at locations in between Australia, Taiwan and the Solomon Islands. *T. sp*. individuals from the western Pacific were reciprocally monophyletic from the individuals from Ningaloo (Indian Ocean) and the single sequence from Taiwan (Fig. 5).

MtDNA genealogies place *T. sp* as sister species to *T. crocea* and *T. squamosa*, with strong support for monophyly of this group of three species (Figs. 2 and 3). *Tridacna maxima* and *T. squamosina* formed a second clade, but with less support across phylogenetic analyses (Fig. 2) probably because only *16S* sequences were available for *T. squamosina*. Monophyly of *T. crocea* and *T. squamosa* was reported in previous mtDNA based phylogenetic analyses [9], [15], but not in allozyme analyses [14] where *T. squamosa* was sister to *T. crocea* and *T. maxima*. Monophyly of the *Chametrachea* subgenus (including *T. squamosina, T. crocea, T. maxima, T. sp.* and *T. squamosa*) [15], [44] was supported in individual gene analyses and the concatenated StarBEAST searches (Fig. 2). Monophyly of the *Tridacna* subgenus (including *T. derasa*, *T. mbalavuana*, and *T. gigas*) was not well supported in any of our mtDNA analyses, with these taxa appearing basal to the *Chametrachea*, but missing and non-overlapping data may have contributed to the low resolution.

Previous phylogeographic studies of *T. crocea* [24], [26], [29] and *T. maxima* [25] from Indonesia show geographic restriction of several clades. The mtDNA gene trees within these papers delineate clusters comprising haplotypes from western Sumatra (Sunda), Wallacea, and northwest New Guinea (Sahul) [24]–[26], [29] with some mixing between clades particularly in the Bird's Head Peninsula of northwest New Guinea [26]. Our samples showed an additional and deeper evolutionary break for *T. crocea* to the east of Cenderawasih Bay, whereby individuals from the Solomon Islands, Torres Strait, and Great Barrier Reef form a monophyletic group and do not share any mtDNA haplotypes with northwest New Guinea or locations in Wallacea (Fig. 5). Therefore, it appears that the distinct clade of *T. crocea* haplotypes from northwest New Guinea (with some spillover westward into Wallacea [26]) is regionally endemic and does not extend into the west Pacific. These patterns are not due to differences in DNA sequencing interpretation between research groups, as samples (from [24], [26], [41]) are mutually consistent and a single *T. crocea* (from [15]) falls within the larger Pacific *T. crocea* clade. Based on present sampling, we can place this newly discovered genetic discontinuity between Cenderawasih Bay and the Solomon Islands in the north and between the Aru Basin and Torres Strait in the south. For *T. maxima*, in contrast, the distinct haplotypes from northwest New Guinea fall in the same clade as west Pacific haplotypes. Thus the northwest New Guinea clade of *T. maxima* can now be viewed as a westward extension of Pacific variants, albeit with no shared haplotypes between locations.

With only two species to compare, we can only speculate as to why the mtDNA patterns differ between species, although greater overall population genetic structure in *T. crocea* compared to *T. maxima* is consistent with previously co-sampled regions (for instance, [24] in comparison to [25]). Because of the diffuse sampling for *T. crocea*, we cannot pinpoint a specific location of geographic differentiation east of Cenderawasih Bay, yet at a macroscale this observation is consistent with mtDNA patterns in a butterflyfish [52], a reef fish [53], and a sea star (Crandall pers. comm.) and may be associated with a long stretch (>700 km) of coastline east of Cenderawasih Bay with sparse reef habitat [54].

In *T. maxima*, we found that Solomon Islands haplotypes cluster with haplotypes from the Great Barrier Reef; this affinity contrasts with allozyme results that show substantial divergence between Solomon Islands and Great Barrier Reef populations [33]. The nature of these differing patterns cannot be explored further as allozyme results are not directly comparable across research groups.

The broadscale geographic and multispecies phylogenetic results of this study, consolidated with those of previous investigations, reveal new aspects of regional patterns and highlight key uncertainties in the current knowledge of *Tridacna*. A common result among population genetic studies of *Tridacna* species to date is that there is substantial population structure. Such genetic differentiation may be due in part to the relatively short planktonic larval duration of approximately 9 days [12] that is likely to restrict dispersal distances. The discovery of an undescribed species adds to other recent species discoveries in *Tridacna* [9]–[11], but the broad distribution of *T. sp.* illustrates that cryptic species can remain undetected even in such conspicuous groups as giant clams.

Both the discovery of a new species and the observation of substantial geographic differentiation are relevant to monitoring of local stocks and human transport of clams. First, the presence of a cryptic sympatric species would result in overestimates of species abundance where clam populations are censused. Second, human-aided movements could cause species to be introduced to regions outside their natural range and, similarly, are likely to introduce foreign genetic material into local populations. *Tridacna maxima*, *T. squamosa*, *T. derasa, T. mbalavuana* and *T. gigas* were frequently translocated during the 1980's and 1990's (some human assisted movements continuing into this century) by governmental, commercial and conservation organizations to combat local depletion and facilitate the live culture trade [55]. Third, depleted populations are unlikely to receive immigrants from geographically distant locations via planktonic dispersal and, therefore, recovery may be slow or negligible even when local harvesting has ceased. Results from giant clams underscore two important themes emerging from genetic investigations of marine organisms: cryptic species are common [19], [20], [56], [57], and many species are genetically heterogeneous across their geographic range [58].

## Supporting Information

Document S1 **Genbank accession numbers for all included sequences.**
(XLS)
